# Dissociated Biologic Responses Across the United Airway: A Case Report of Severe Asthma With Chronic Rhinosinusitis With Nasal Polyps and Eosinophilic Otitis Media

**DOI:** 10.1002/rcr2.70715

**Published:** 2026-08-06

**Authors:** Tomoko Tajiri, Naoki Takemoto, Toshiya Minakata, Motohiko Suzuki, Yoshiyuki Ozawa, Hirotsugu Ohkubo

**Affiliations:** ^1^ Department of Respiratory Medicine, Allergy and Clinical Immunology Nagoya City University Graduate School of Medical Sciences Nagoya Japan; ^2^ Department of Otorhinolaryngology Head and Neck Surgery Nagoya City University Graduate School of Medical Sciences Nagoya Japan; ^3^ Department of Diagnostic Radiology Fujita Health University School of Medicine Toyoake Japan

**Keywords:** asthma, biologic switching, chronic rhinosinusitis with nasal polyps, eosinophilic otitis media, type 2 inflammation

## Abstract

Asthma, chronic rhinosinusitis with nasal polyps (CRSwNP), and eosinophilic otitis media (EOM) share type 2 inflammatory pathways. Although type 2‐targeted biologic therapies have shown efficacy for these diseases, some patients exhibit dissociated responses to a single biologic. A case of severe asthma with CRSwNP and EOM that showed dissociated responses to biologic therapies and was successfully managed by biologic switching is reported. A 55‐year‐old man with asthma, recurrent CRSwNP requiring multiple surgeries and oral corticosteroid‐dependent EOM was referred to our hospital. Nineteen months after starting dupilumab, EOM and endoscopic and radiological findings of CRSwNP improved partially. However, his nasal symptoms and olfactory dysfunction persisted, and asthma remained uncontrolled. After switching from dupilumab to mepolizumab, asthma control, nasal symptoms and olfactory dysfunction improved, and EOM remained well controlled. Careful monitoring of disease activity and consideration of biologic switching may help achieve optimal control in patients with type 2 united airway diseases.

## Graphical abstract

We report a case of severe asthma with chronic rhinosinusitis with nasal polyps and eosinophilic otitis media that was successfully controlled by switching biologic therapy from dupilumab to mepolizumab. Careful monitoring of disease activity and consideration of biologic switching may help achieve optimal control with currently available biologics, and future dual‐blockade strategies may further improve outcomes in selected patients with type 2 united airway diseases.

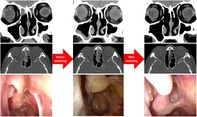

## 1 Introduction

Asthma and chronic rhinosinusitis with nasal polyps (CRSwNP) frequently coexist under the concept of a ‘united airway’. Eosinophilic otitis media (EOM) is also associated with asthma and CRSwNP and is linked to more severe disease [1]. These conditions are driven by type 2 inflammation; therefore, biologics targeting these pathways are effective [1]. Several studies [1, 2] have reported biologic efficacy in asthma with comorbid CRSwNP and EOM. In some cases, biologic switching has been performed for optimal control because responses differ among these conditions [2, 3]. Most previous reports [2, 3] have described switching from other biologics to dupilumab, whereas data regarding switching from dupilumab to other biologics remain limited.

A case of severe asthma with CRSwNP and EOM successfully controlled by switching biologic therapy from dupilumab to mepolizumab is presented.

## 2 Case Report

A 55‐year‐old man was diagnosed with asthma at the age of 32 years and was treated with an inhaled corticosteroid/long‐acting β_2_‐agonist (ICS/LABA; fluticasone furoate/vilanterol 200/25 μg). He did not have aspirin hypersensitivity. At the age of 36 years, he underwent bilateral endoscopic sinus surgery (ESS) for CRSwNP at another hospital. At the age of 38 years, a recurrent left nasal polyp was removed by polypectomy. At the age of 49 years, CRSwNP recurred again, necessitating revision bilateral ESS.

At the age of 50 years, he developed EOM that met the diagnostic criteria [1]. A tympanostomy tube was inserted in the right ear, and he was treated with oral corticosteroids (OCS) and a leukotriene receptor antagonist for 2 years. At the age of 53 years, left‐sided EOM recurred with aural fullness and conductive hearing loss. Following a 1‐week course of OCS, he relocated for work and discontinued ICS/LABA. Despite OCS treatment, his EOM symptoms persisted and CRSwNP symptoms, including rhinorrhea and anosmia, recurred. He was referred to the Department of Otorhinolaryngology at our hospital for refractory EOM and CRSwNP.

**FIGURE 1:**
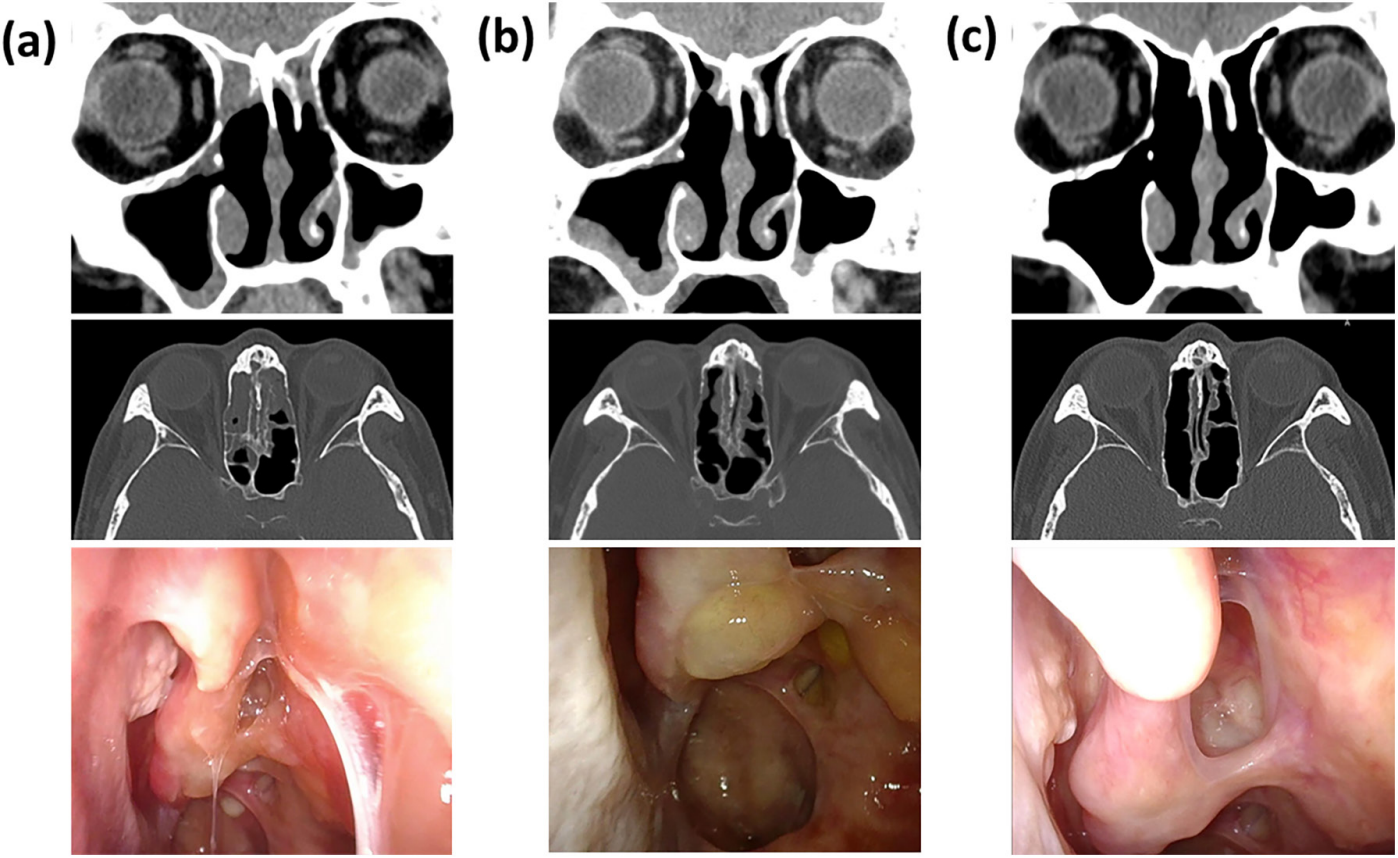
Paranasal sinus computed tomography images and endoscopic views of the left nasal cavity obtained (a) before dupilumab administration, (b) at the time of switching from dupilumab to mepolizumab and (c) 10 months after mepolizumab administration.

At presentation, otoscopy showed an opaque tympanic membrane with middle‐ear effusion. Pure‐tone audiometry demonstrated hearing thresholds of 18.8 dB (right) and 21.3 dB (left), with left‐sided conductive hearing loss. His nasal symptoms had worsened, with a 22‐item Sinonasal Outcome Test (SNOT‐22) score of 44. The nasal polyp score (NPS) was 6, the Lund‐Mackay score (LMS) was 9 (Figure 1) and he had complete anosmia, with an Open Essence (OE; Fujifilm, Osaka, Japan), a card‐based olfactory identification test consisting of 12 odour cards, score of 0. Fractional exhaled nitric oxide was 3.7 ppb, and the blood eosinophil count was 409/μL (Table 1). Serum total immunoglobulin E (IgE) was 212 IU/mL, and allergen‐specific IgE was positive for house dust and Japanese cedar. Myeloperoxidase‐antineutrophil cytoplasmic antibody was not assessed. Dupilumab (300 mg every 2 weeks) was started for severe refractory CRSwNP.

**TABLE 1:** Longitudinal changes in otologic, sinonasal and respiratory variables across biologic switching.

Variables	Dupilumab			Mepolizumab			
	0 M	1 M	19 M	0 M	1 M	6 M	10 M
4‐Frequency PTA (dB)							
Right ear	18.8	11.3	13.8		15.0	12.5	NE
Left ear	21.3	10.0	12.5		13.8	11.3	NE
Opaque tympanic membrane with middle‐ear effusion	Yes	No	No		No	No	No
Nasal polyp score	6	2	3		2	1	0
Lund–Mackay score	9	NE	4		NE	3	1
Open essence	0	NE	3		6	8	9
SNOT‐22							
Total	44	44	49		15	43	25
Nasal	2.1	2.1	2.5		0.6	2.5	0.6
Ear/facial	1.5	1.3	1.3		0	1.3	0.5
Sleep	1.3	1.5	1.8		0.8	1.5	1.3
Function	3.3	3.3	3.3		1.7	2.3	2.3
Emotion	2.0	2.0	2.3		0.7	1.7	2.0
FeNO, ppb	3.7	NE	10		10.7	6	8.3
FEV_1_, %predicted	107	NE	110.5		107.5	107.5	105.9
ACQ‐7 score	2.6	NE	1.6		1.3	0.9	0.4
Cough VAS score	76	NE	46		49	41	3
Blood eosinophils (μL)	409	265	339		9	8	11

Abbreviations: ACQ‐7, the 7‐item Asthma Control Questionnaire; CRSwNP, chronic rhinosinusitis with nasal polyps; EOM, eosinophilic otitis media; FeNO, Fractional exhaled nitric oxide; FEV_1_, forced expiratory volume in 1 s; M, month; NE, not evaluated; PTA, pure‐tone average; SNOT‐22, the 22‐item Sinonasal Outcome Test; VAS, visual analog scale.

One month after starting dupilumab, his aural fullness and hearing loss improved. Otoscopy demonstrated resolution of the tympanic membrane opacity and middle‐ear effusion, and his pure‐tone audiometry findings improved (Table 1). However, rhinorrhea and olfactory dysfunction persisted, and asthma worsened. Five months after starting dupilumab, he was referred to Respiratory Medicine because of uncontrolled asthma (a 7‐item Asthma Control Questionnaire (ACQ‐7) score, 2.6). Treatment with ICS/LABA (fluticasone furoate/vilanterol 200/25 μg) was resumed, and a long‐acting muscarinic antagonist and theophylline were added. Although EOM improved and the NPS and LMS decreased to 3 and 4, respectively, his asthma and CRSwNP symptoms persisted, including cough, rhinorrhea and olfactory dysfunction (Table 1). Nineteen months after starting dupilumab, biologic therapy was switched to mepolizumab because of uncontrolled severe asthma (ACQ‐7 score, 1.6) and CRSwNP (SNOT‐22 score, 49), persistent peripheral eosinophilia and ongoing symptoms, including cough (cough Visual Analog Scale (VAS) score, 46) and olfactory dysfunction (OE score, 3). No adverse events occurred prior to switching to mepolizumab.

Ten months after switching to mepolizumab, the patient's cough subsided (cough VAS score, 3), asthma control improved (ACQ‐7 score, 0.4), CRSwNP symptoms improved (SNOT‐22 score, 25), olfactory dysfunction recovered (OE score, 9) and the NPS and LMS decreased to 0 and 1 (Figure 1), respectively. EOM remained well controlled (Table 1).

## 3 Discussion

A case of severe asthma with comorbid CRSwNP and EOM that was successfully managed by switching biologics from dupilumab to mepolizumab was presented. This case highlights dissociated biologic responses across united airway diseases and the utility of biologic switching when disease control is not achieved.

Asthma, CRSwNP and EOM share type 2 inflammatory pathways [4]. Although type 2‐targeted biologics are effective, some patients exhibit discordant responses, necessitating biologic switching to achieve comprehensive disease control [3]. Most reported cases [2, 3] involving this triad have described switching from omalizumab, mepolizumab or benralizumab to dupilumab, whereas successful switching from dupilumab to mepolizumab was seen in the present case. Although systematic reviews and network meta‐analyses ranked dupilumab highest for CRSwNP outcomes [3], these findings are not always applicable to real‐world clinical practice.

In the present case, dupilumab improved EOM and partially improved objective CRSwNP findings. However, CRSwNP symptoms, including rhinorrhea and olfactory dysfunction, and asthma remained uncontrolled. The persistence of sinonasal symptoms despite improvement in objective sinonasal findings during dupilumab treatment may be partly explained by impaired sleep, emotional well‐being and functional status associated with uncontrolled asthma, based on serial changes in the five SNOT‐22 domain scores (Table 1). After switching to mepolizumab, CRSwNP symptoms and asthma control improved. EOM remained well controlled. The dramatic improvement in the SNOT‐22 score appeared to reflect both improvement in sinonasal symptoms and amelioration of the systemic burden associated with better asthma control. These findings suggest that responses to biologic therapies may differ among different organ manifestations of united airway disease. A previous study [3] described patients with severe asthma and CRSwNP who switched biologics due to insufficient control of either upper or lower airway disease.

The mechanisms underlying these differential responses remain unclear. Although type 2 inflammatory pathways contribute to the pathogenesis of asthma [4], CRSwNP [4] and EOM [1], the relative contributions of IL‐4/IL‐13‐, IL‐5/eosinophil‐ and IgE‐mediated inflammatory pathways may differ among organs. In EOM, both IL‐5/eosinophil‐mediated inflammation and IL‐4/IL‐13‐driven tissue responses may play important roles. A previous study [5] demonstrated that the expressions of eosinophil‐derived neurotoxin, IL‐4 and IL‐5 were elevated in the middle‐ear effusions from patients with EOM, suggesting that both pathways contribute to EOM pathogenesis. In contrast, CRSwNP is characterized by tissue and blood eosinophilia and elevated type 2 inflammatory mediators. In patients with a predominantly eosinophil‐driven phenotype, inhibition of the IL‐5/eosinophil pathway may result in substantial clinical benefit. In the present case, baseline blood eosinophilia and relatively low fractional exhaled nitric oxide levels may suggest that eosinophil‐mediated inflammation contributed substantially to CRSwNP and lower airway disease, although direct endotypic assessment was not performed. Another possible explanation is that the inflammatory milieu may evolve over time through recurrence, surgery and OCS use, potentially altering the relative contribution of the inflammatory pathways. Indeed, individual variability in inflammatory endotypes has been reported in asthma [6] and CRSwNP [4].

Given the heterogeneous inflammatory endotypes, future therapeutic strategies involving simultaneous targeting of multiple type 2 inflammatory pathways, such as bispecific antibodies targeting multiple cytokines, may provide more comprehensive control of inflammation across different organs within the united airway. Although such approaches remain investigational, they may represent a promising therapeutic option for selected patients showing discordant responses across different organs when treated with a single biologic.

This case report has several limitations. Although spirometry was performed, no improvement in lung function was observed after the biologic switch. Furthermore, neither chest computed tomography nor airway hyperresponsiveness testing was performed.

In conclusion, this case suggests dissociated biologic responses across united airway diseases. Careful monitoring and consideration of biologic switching may help achieve optimal disease control with currently available biologics, and future dual‐blockade strategies may further improve outcomes in selected patients with type 2 united airway diseases.

## Author Contributions

Conceptualisation: Tomoko Tajiri. Data collection: Tomoko Tajiri, Naoki Takemoto and Toshiya Minakata. Radiological assessment: Yoshiyuki Ozawa. Writing – original draft preparation: Tomoko Tajiri. Writing – review and editing: Naoki Takemoto and Motohiko Suzuki. Supervision: Hirotsugu Ohkubo. All authors have read and agreed to the published version of the manuscript.

## Ethics Statement

This study was conducted in accordance with the principles of the Declaration of Helsinki. This case report was approved by the ethics committee of Nagoya City University (60‐24‐0185).

## Consent

The authors declare that written informed consent was obtained for the publication of this manuscript and accompanying images and attest that the form used to obtain consent from the patient complies with the Journal requirements as outlined in the author guidelines.

## Conflicts of Interest

Yoshiyuki Ozawa is an Editorial Board member of Respirology Case Reports and a co‐author of this article. He was excluded from all editorial decision‐making related to the acceptance of this article for publication. The other authors declare no conflicts of interest.

## Data Availability

The data that support the findings of this study are available from the corresponding author upon reasonable request.
